# The chromodomain helicase CHD4 regulates ERBB2 signaling pathway and autophagy in ERBB2^+^ breast cancer cells

**DOI:** 10.1242/bio.038323

**Published:** 2019-04-09

**Authors:** Carolina D'Alesio, Grazia Bellese, Maria Cristina Gagliani, Anastasia Lechiara, Martina Dameri, Elena Grasselli, Luisa Lanfrancone, Katia Cortese, Patrizio Castagnola

**Affiliations:** 1 DIMES, Department of Experimental Medicine, Human Anatomy, Università di Genova, Via Antonio de Toni 14, 16132, Genova, Italy; 2 DIMI, Department of Internal Medicine, Pharmacology, Università di Genova, Viale Benedetto XV, 16132, Genova, Italy; 3 DISTAV, Department of Earth, Environment and Life science, Università di Genova, Corso Europa 26, 16132, Genova, Italy; 4 Department of Experimental Oncology, European Institute of Oncology, Via Adamello 16, 20139, Milano, Italy; 5 IRCCS Ospedale Policlinico San Martino, Largo Rosanna Benzi 10, 16132, Genova, Italy

**Keywords:** Breast cancer, ERBB2, CHD4, Trastuzumab, Autophagy

## Abstract

The chromodomain helicase DNA-binding 4 (CHD4), a member of the nucleosome remodeling and deacetylases (NuRD) complex, has been identified as an oncogene that modulates proliferation and migration of breast cancers (BC). ERBB2 is an oncogenic driver in 20–30% of BC in which its overexpression leads to increased chemoresistance. Here we investigated whether CHD4 depletion affects the ERBB2 cascade and autophagy, which represents a mechanism of resistance against Trastuzumab (Tz), a therapeutic anti-ERBB2 antibody. We show that CHD4 depletion in two ERBB2^+^ BC cell lines strongly inhibits cell proliferation, induces p27^KIP1^ upregulation, Tyr^1248^ ERBB2 phosphorylation, ERK1/2 and AKT dephosphorylation, and downregulation of both ERBB2 and PI3K levels. Moreover, CHD4 silencing impairs late stages of autophagy, resulting in increased levels of LC3 II and SQSTM1/p62, lysosomal enlargement and accumulation of autolysosomes (ALs). Importantly, we show that CHD4 depletion and concomitant treatment with Tz prevent cell proliferation *in vitro*. Our results suggest that CHD4 plays a critical role in modulating cell proliferation, ERBB2 signaling cascade and autophagy and provide new insights on CHD4 as a potential target for the treatment of ERBB2^+^ BC.

## Abstract (precis)

**Summary:** Inhibition of CHD4 expression impairs cell proliferation and survival through downregulation of ERBB2 signaling and block of autophagy. Therefore, CHD4 should be considered a potential target in ERBB2^+^ breast cancers.

## INTRODUCTION

Breast cancer (BC) is considered a collection of diseases showing heterogeneity at molecular, histopathological and clinical level, which generates variable clinical courses and responses to treatments (Polyak, 2011). The genetic and molecular characterization of breast tumors has allowed the identification of five main subtypes according to the receptor status (estrogen, progesterone or ERBB2) (Goldhirsch et al., 2011). Among them, the *ERBB2* overexpressing (ERBB2^+^) subtype is characterized by amplification or overexpression of the *ERBB2* (*ERBB2/Neu*) oncogene and accounts for approximately 20–30% of all BCs (Yarden, 2001a). ERBB2 belongs to the human epidermal growth factor receptor (EGFR) family, which consists of four members (ERBB1/EGFR, ERBB2, ERBB3 and ERBB4). Of the four ERBB receptors, only ERBB2 has no known ligand and is subjected to an additional layer of regulation mediated by the molecular chaperone HSP90 (Castagnola et al., 2016; Bertelsen and Stang, 2014; Miyata et al., 2013). Several malignancies are associated with mutations or increased expression of members of the EGFR family, including lung, breast, stomach, colorectal, head and neck, thyroid, pancreatic carcinomas and glioblastoma (Yarden, 2001b; Li et al., 2018; Minuto et al., 2018; Sigismund et al., 2018; von Achenbach et al., 2018; Rodríguez-Antona et al., 2010). The ERBB receptors work as homo- or heterodimers able to engage different downstream signaling modules, such as Ras/Raf/MAPK and phosphatidylinositol 3-kinase (PI3K)/AKT pathways (Harari and Yarden, 2000; Carmona et al., 2016; Bagnato et al., 2017). In addition, *ERBB2* overexpression correlates with increased progression through the cell cycle by affecting CDKN1A/p21^WAF1^ and CDKN1B/p27^KIP1^ (Carmona et al., 2016).

Trastuzumab (Tz) is an inhibitory monoclonal antibody that targets the extracellular domain of ERBB2 and is used as a front-line therapy for the treatment of ERBB2^+^ BCs. Tz downregulates the downstream PI3K/AKT and Ras/Raf/MEK/ERK1/2 signaling cascade, resulting in the impairment of cell proliferation (Yakes et al., 2002; Vu and Claret, 2012). Moreover, ERBB2 endocytic downregulation, cell cycle arrest in G1 phase and nuclear accumulation of the cell cycle inhibitor p27^KIP1^ have been reported (Valabrega et al., 2005; Nahta and Esteva, 2006; Le et al., 2005). Combinations of Tz with chemotherapeutic agents or other targeted inhibitors has reduced recurrence rates, improved outcome and prolonged the survival of patients; however, *de novo* and acquired resistance to Tz are still frequently observed (Nahta and Esteva, 2006; Lavaud and Andre, 2014; Di Modica et al., 2017).

The catabolic process of autophagy is a protein degradation process regulated by the mTOR-signaling pathway, which degrades cytoplasmic constituents within lysosomes (Yin et al., 2016). In cancer biology, autophagy has emerged as a resistance mechanism to multiple anticancer therapies such as kinase inhibitors or chemotherapy (Amaravadi et al., 2011). Protective autophagy might be induced in BC cells treated with anti-ERBB2 drugs such as Lapatinib or Tz, allowing cancer cells to survive (Chen et al., 2016; Vazquez-Martin et al., 2009). For these reasons, autophagy inhibitors are under intense investigations as novel anti-cancer agents (Amaravadi et al., 2011; Bortnik and Gorski, 2017). Recently, we demonstrated that the diterpene carnosic acid (CA) in combination with Tz impairs late autophagy, partially restoring Tz sensitivity in Tz-resistant cells (D'Alesio et al., 2017).

The chromatin remodeling helicase CHD4, a component of the nucleosome remodeling and deacetylases (NuRD) complex, has been recently identified as an essential regulator of BC growth in murine and patient derived xenograft (PDX) BCs (D'Alesio et al., 2016) and correlates with poor prognosis in cancers (Nio et al., 2015; Xia et al., 2017). In addition to its role in transcriptional regulation, *CHD4* is also implicated in DNA damage response, cell cycle progression (O'Shaughnessy and Hendrich, 2013), cell stemness in a model of hepatocellular carcinoma (Nio et al., 2015) and in organogenesis and postnatal organ/tissue differentiation (Gómez-Del Arco et al., 2016). In a triple negative BC cell line, *CHD4* depletion causes a significant reduction of cell proliferation and migration *in vitro* and a dramatic decrease of the tumor mass *in vivo* (D'Alesio et al., 2016). This inhibition was also found in luminal B and triple negative PDX models and in a transgenic mouse model (MMTV/*Neu*T) having the rat *ERBB2* ortholog activated (D'Alesio et al., 2016). Moreover, *CHD4* regulates BC cell cycle progression and its silencing determines the accumulation of cells in the G0 phase, a dramatic reduction of DNA synthesis, together with an upregulation of p21^WAF1^ (D'Alesio et al., 2016). Most importantly, the depletion of *CHD4* in MCF10A cells, a human mammary epithelial cell line that lacks tumorigenic potential, did not affect cell proliferation and migration *in vitro*, suggesting that *CHD4* targeting has the potential to become a novel therapeutic strategy to impair BC progression (D'Alesio et al., 2016).

Interestingly, evidence shows that the NuRD complex plays a role in the epigenetic regulation of autophagy. It has been demonstrated that repression of *mTOR* expression by *SOX2* promotes cellular reprogramming and induction of autophagy through the recruitment of the NuRD complex (Wang et al., 2013). In addition, the methyltransferase EZH2 represses the expression of mTOR pathway-related genes via the NuRD complex component MTA2 (metastasis associated 1 family, member 2) (Wei et al., 2015).

In this work, we aimed at filling the gap of knowledge about the role of *CHD4* in the specific regulation of the ERBB2-mediated signaling cascades and autophagy in ERBB2^+^ BC cells. We have found that *CHD4* depletion impairs ERBB2 molecular pathways downregulating the phosphorylation status of pAKT and pERK. In addition, we demonstrated that *CHD4* silencing impairs late stages of autophagy likely contributing to the impairment of BC cell proliferation. Lastly, we showed that *CHD4* deprivation cooperates with Tz in zeroing ERBB2^+^ BC cell proliferation. Our work provides new insights on *CHD4* as a potential target for the treatment of ERBB2^+^ BC to be used alone or in combination with traditional anticancer agents.

## RESULTS

### *CHD4* regulates ERBB2^+^ BC cell growth

**Fig. 1.**
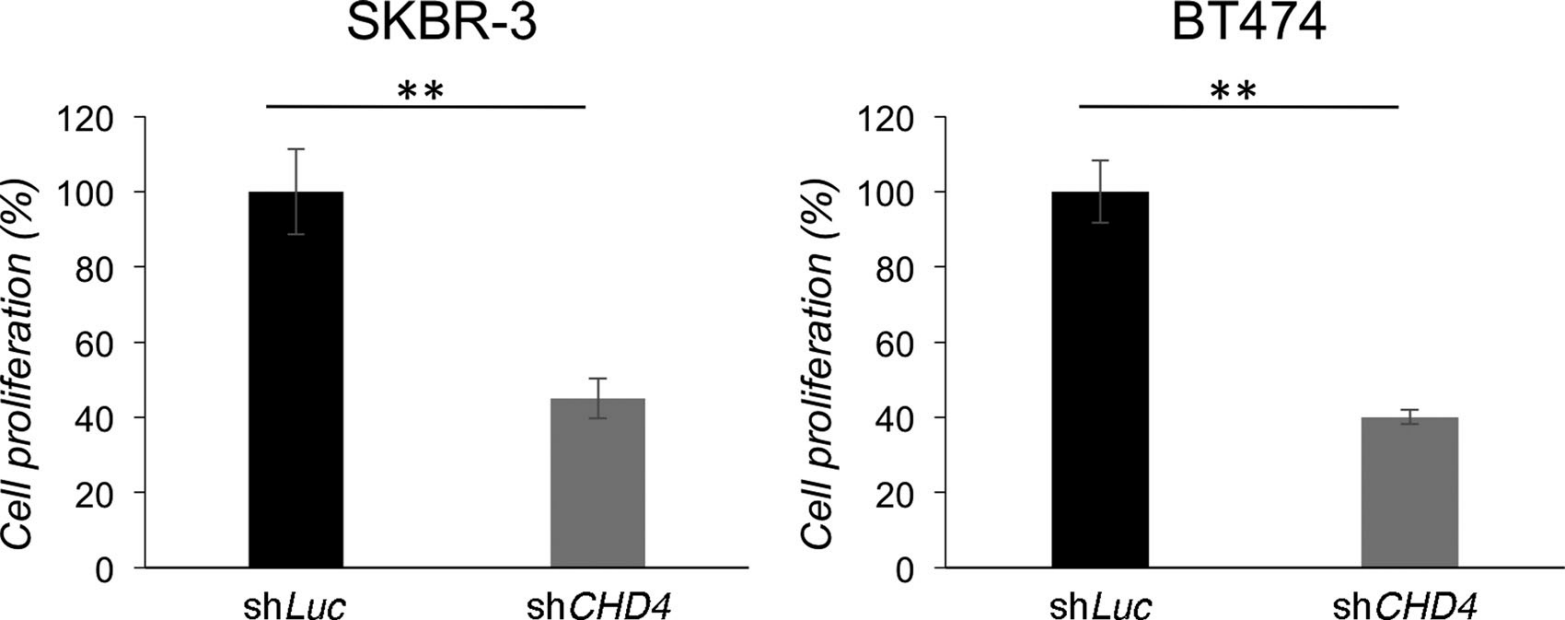
***CHD4* depletion inhibits *in vitro* proliferation of ERBB2^+^ breast cancer cells.** SKBR-3 and BT474 cells transduced with sh*CHD4* or control sh*Luc* were cultured for 7 days. Cell proliferation is expressed as percentage of the maximum absorbance (at 570 nm) value obtained after exposure of cultures to MTT for 4 h. Mean values and s.d. (indicated as vertical bars) from three independent replicates are shown. **P<0.01.

As the helicase CHD4 is implicated in the development of murine ERBB2^+^ BC (D'Alesio et al., 2016) we wanted to establish its role in a human ERBB2^+^ BC cell model. To this end, we used SKBR-3 (estrogen and progesterone receptors negative) and BT474 (estrogen and progesterone receptors positive) cell lines. In particular, we transduced SKBR-3 and BT474 cells with two pooled shRNAs targeting *CHD4* (sh*CHD4*) or control vector (sh*Luc*) for 72 h and evaluated cell survival by MTT analysis after 7 days. As shown in Fig. 1, loss of *CHD4* determined a statistically significant inhibition of ERBB2^+^ BC cell proliferation *in vitro*, compared to control population. These data confirmed that *CHD4* silencing is effective in the inhibition of survival of ERBB2^+^ BC cells *in vitro*.

### *CHD4* depletion inhibits ERBB2 signaling pathway

**Fig. 2.**
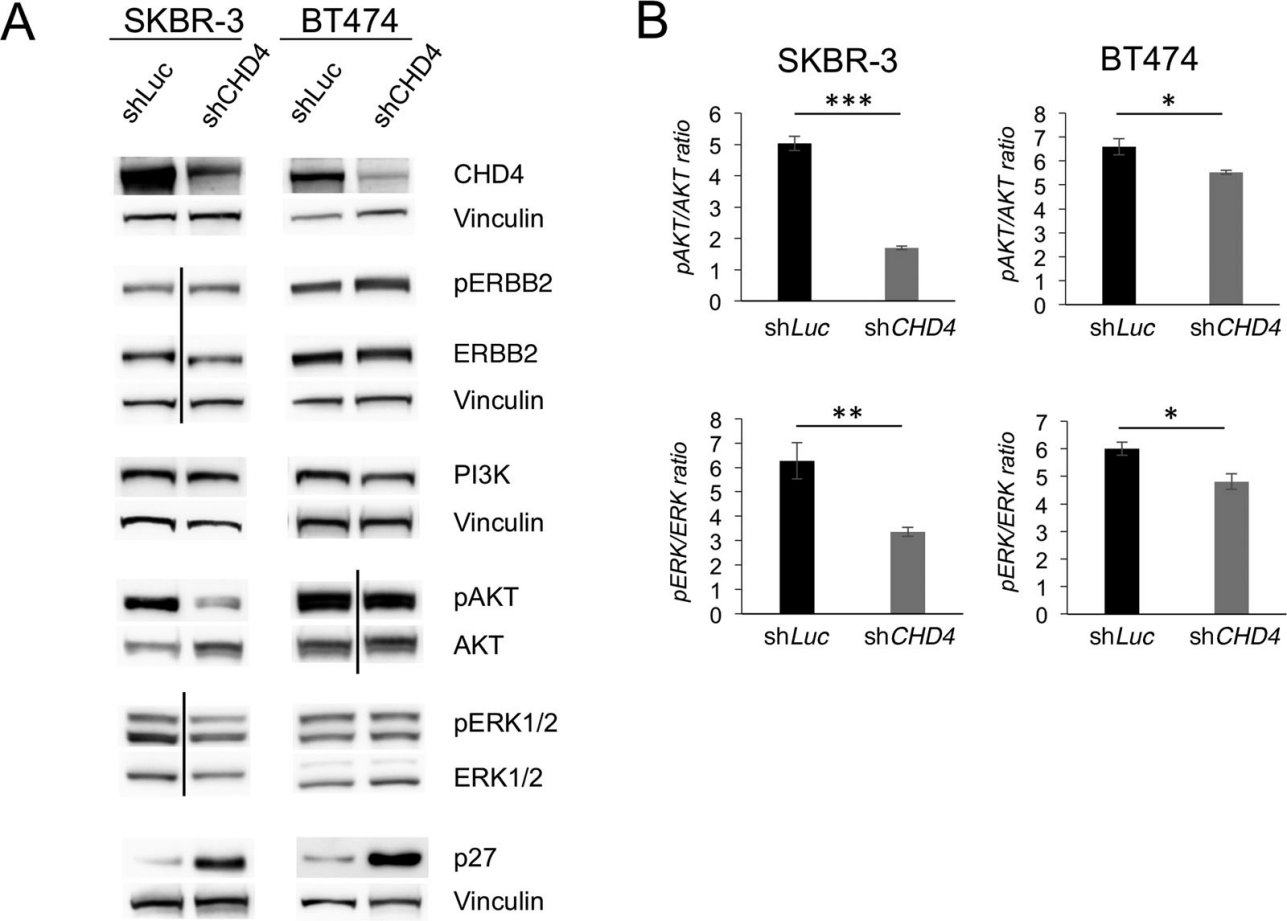
**Loss of *CHD4* inhibits HER2** **signaling in SKBR-3 and BT474 cells.** ERBB2^+^ BC cells transduced with sh*CHD4* or control sh*Luc* were cultured for 48 h and subsequently lysed. (A) The immunoblot analysis was performed with anti-CHD4, anti-ERBB2, anti-phospho-Tyr^1248^ ERBB2, anti-phospho-Ser^473^ AKT, anti-AKT, anti-phospho-Thr^202^/Tyr^204^ ERK1/2, anti-ERK1/2, anti-p27^kip1^ and anti-Vinculin. Vinculin is used as loading control. Representative immunoblot images from three independent replicates. Vertical lines indicate non-contiguous bands obtained from samples run on the same blot. (B) Histograms represent the ratios of phospho-Ser^473^ AKT/AKT and anti-phspho-Thr^202^/Tyr^204^ ERK1/2/ ERK1/2 performed with Alphaplex assay. Mean values and s.d. (indicated as vertical bars) from three replicates are shown. *P<0.05, **P<0.01, ***P<0.001.

As ERBB2^+^ BC cells heavily depend on ERBB*2* receptor signaling for their growth and survival, and because *CHD4* depletion inhibits BC tumor development in the *MMTV/NeuT* model, we hypothesized that *CHD4* silencing might impair the ERBB2 signaling pathway. To this end, we transduced SKBR-3 and BT474 cells with sh*CHD4* or sh*Luc* and examined by immunoblot analysis the major players of the ERBB2 signaling cascade. Upon *CHD4* silencing, we found that ERBB2 phosphorylation is increased on Tyr^1248^, while ERBB2 receptor total levels are only slightly reduced in both cell lines (Fig. 2A and Table S1). To assess whether this decrease in ERBB2 levels was the result of a transcriptional regulation or mRNA degradation, we measured *ERBB2* mRNA levels by qPCR analysis. The result suggested that the minor changes observed in ERBB2 levels are likely due to protein degradation, as the mRNA levels did not decrease but, instead, slightly increased after *CHD4* silencing (Fig. S2).

Furthermore, the evaluation of ERBB2 downstream signaling revealed a downregulation of PI3K protein levels in *CHD4* silenced cells compared to sh*Luc* cells. Remarkably, we also observed a strong dephosphorylation of AKT Ser^473^ in SKBR-3 cells, which was less pronounced in BT474 cells, along with a dephosphorylation of ERK1/2 Thr^202^/Tyr^204^ (Fig. 2A and Table S1). In particular, we measured pAKT/AKT and pERK1/2/ERK ratio also by the Alphaplex assay (see Materials and Methods), which confirmed the immunoblot results (Fig. 2B). Next, we evaluated the p27^kip1^ protein levels, the last member of the ERBB2 signaling cascade. The immunoblot analysis unveiled a strong upregulation of p27^kip1^ levels in *CHD4* silenced cells in both cell lines (Fig. 2A and Table S1). Taken together, these results showed that *CHD4* regulates ERBB2 levels and its signaling cascade in ERBB2^+^ BC cells.

### *CHD4* silencing impairs late autophagy

**Fig. 3.**
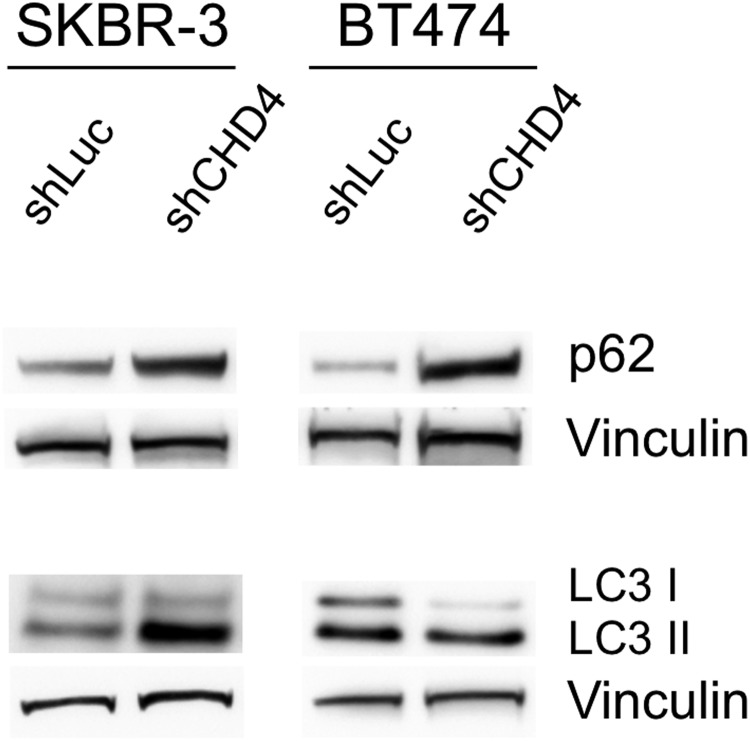
***CHD4* silencing upregulates p62 levels and LC3II/LC3I ratios in ERBB2^+^ BC cells.** SKBR-3 and BT474 cells infected with sh*CHD4* or control sh*Luc* were cultured for 48 h and subsequently lysed. The immunoblot analysis was performed with anti-p62, anti-LC3 and anti-Vinculin (used as loading control). Representative immunoblot images from three independent replicates. Please note that the same blot of SKBR-3 samples probed with the P27 antibody and shown in Fig. 2A was also probed with the P62 antibody shown in this figure and, therefore, their Vinculin normalization signals are identical. For the same reason, the Vinculin normalization signals are identical for the pERBB2/ERBB2 signals shown in Fig. 2A and for the LC3 signals in BT474 samples shown in this figure.

**Fig. 4.**
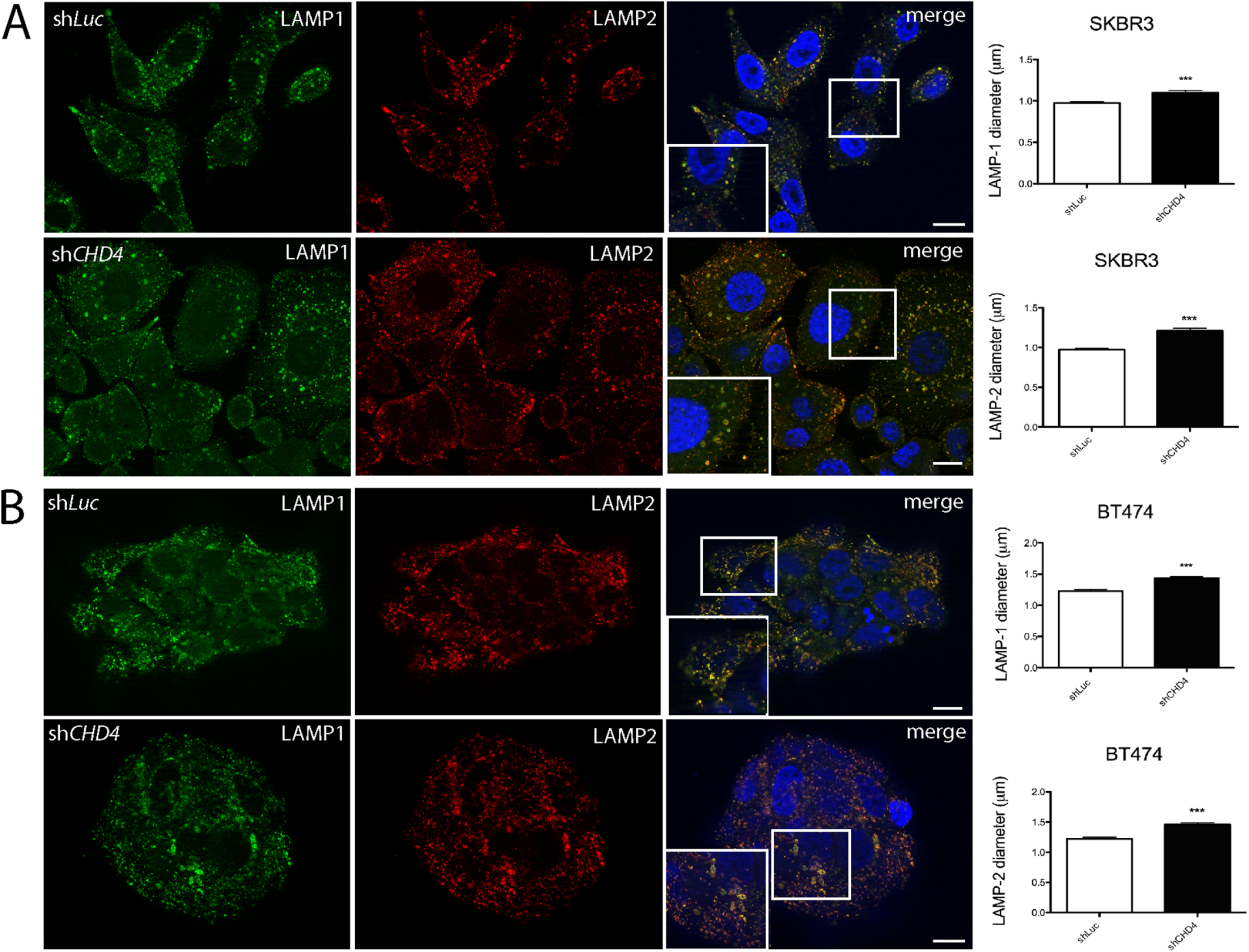
***CHD4* silencing affects lysosomal morphology in SKBR-3 and BT474 cells.** Representative de-convoluted images of sh*Luc* and sh*CHD4* depleted SKBR-3 (A) and BT474 (B) cells. Cells were fixed, permeabilized and incubated with anti-LAMP1 and anti-LAMP2 antibodies to detect lysosomes and Alexa488-conjugated anti-mouse secondary antibody (green signal, LAMP1) and Alexa546-conjugated anti-mouse secondary antibody (red signal, LAMP2). Nuclei were stained with DAPI (blue signal). In *CHD4* silenced cells, lysosomes (LAMP-1 and LAMP-2 positive) appear larger compared to control, sh*Luc* cells. Scale bars: 20 µm. Analysis of LAMP1 and LAMP2 lysosome size was performed on three independent experiments measuring 50 cells in sh*Luc* and sh*CHD4* SKBR-3 and BT474 cells by using Huygens Professional software. Mean values and s.d. of LAMP1 and LAMP2 lysosomes are shown as histograms. ***P<0.001

**Fig. 5.**
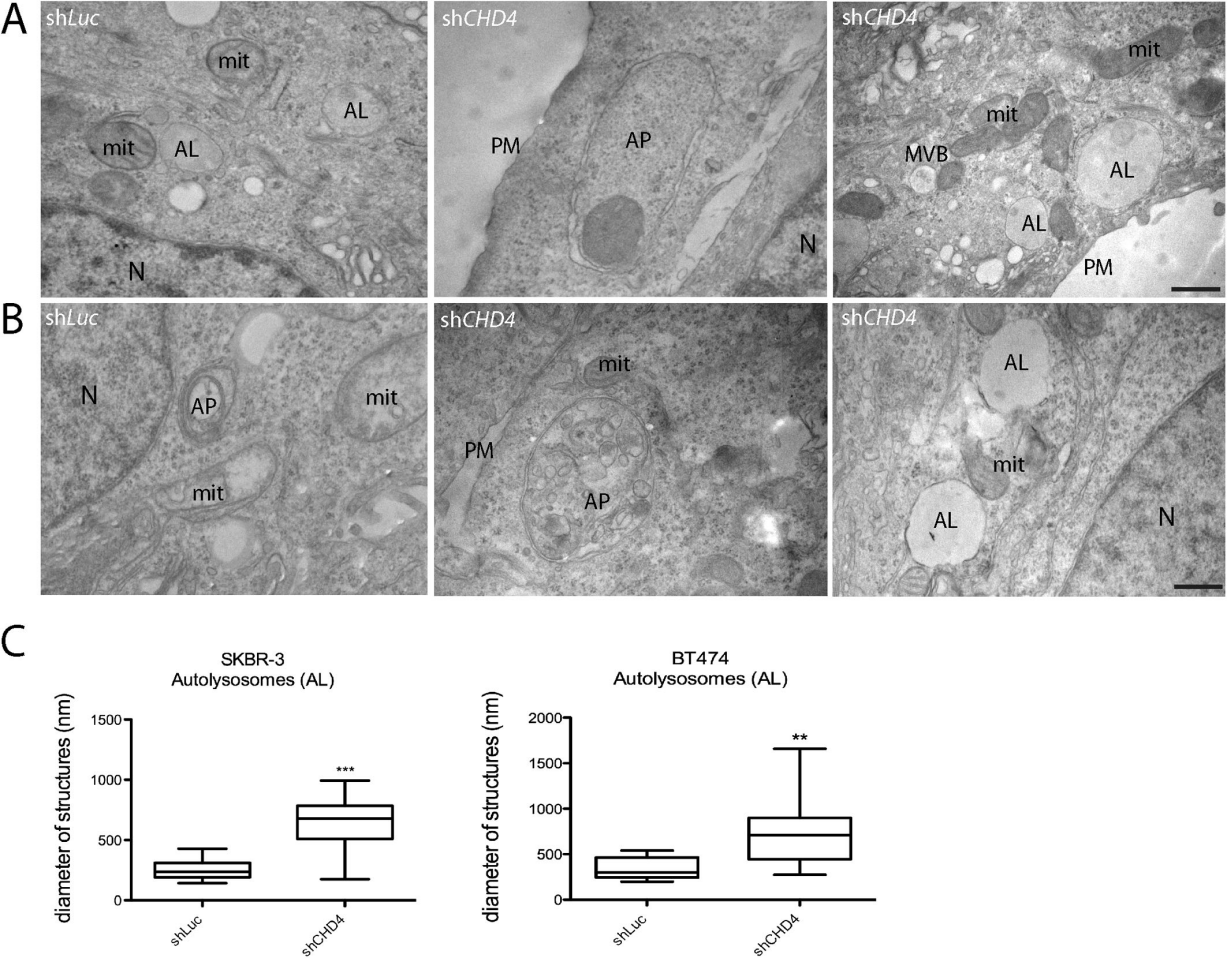
***CHD4* silencing impairs late autophagy accumulating autolysosomes in SKBR-3 and BT474 cells.** Representative TEM images of SKBR-3 (A) and BT474 cells (B) transduced with sh*Luc* or sh*CHD4* for 72 h. Six major categories of structures were identified by morphological criteria in both sh*Luc* and sh*CHD4* cells: multivescicular bodies (MVBs), double-membrane autophagosomes (AP), autolysosomes (AL), nuclei (N), mitochondria (mit), plasma membrane (PM). Scale bars: 500 nm. (C) Box plots showing the AL diameter measured for each experimental condition. For this analysis, 10 whole cells were scored and measured for AL with iTEM imaging software. Note that in *CHD4* depleted cells the diameter of ALs is significantly increased with respect to sh*Luc* control cells, **P<0.01, ***P<0.001 for BT474 and SKBR-3, respectively.

As previous studies showed that the NuRD complex plays a role in the epigenetic regulation of autophagy (Wei et al., 2015; Wang et al., 2013), we hypothesized that *CHD4* silencing might impair this pathway, thus contributing to the growth inhibition of ERBB2^+^ BC cells. To evaluate protein levels of LC3 and p62, the hallmarks of autophagy (Bjørkøy et al., 2009; Menzies et al., 2012), 72 h after transduction cell lysates were prepared and processed by immunoblot analysis. We observed an upregulation of LC3II/LC3I ratio and accumulation of p62 protein levels when *CHD4* was silenced in both SKBR-3 and BT474 cells (Fig. 3 and Table S1). These results suggested that lack of *CHD4* blocks late stages of autophagy which might impair the degradation of both LC3II and p62. To better characterize the effect of *CHD4* inhibition on the autophagy process, we analyzed the autophagic and lysosomal compartments by immunofluorescence and ultrastructural analysis. In particular, by immunofluorescence analysis, we evaluated LAMP1 and LAMP2 positive lysosomes and measured their size. We found that *CHD4* silencing caused a slight but statistically significant increase of the diameter of these organelles (Fig. 4), which is consistent with an increase of the size of autolysosomes due to a block of late autophagy. To gain high resolution information on the ultrastructure of autophagic organelles in our cell model system, we performed a morphological electron microscopy analysis. Importantly, we found the presence of double membrane autophagosomes (AP) and a significant enlargement of autolysosomal structures (AL) in *CHD4* silenced cells compared to controls (Fig. 5).

### Loss of *CHD4* cooperates with Tz in inhibiting proliferation of ERBB2^+^ BC cells

**Fig. 6.**
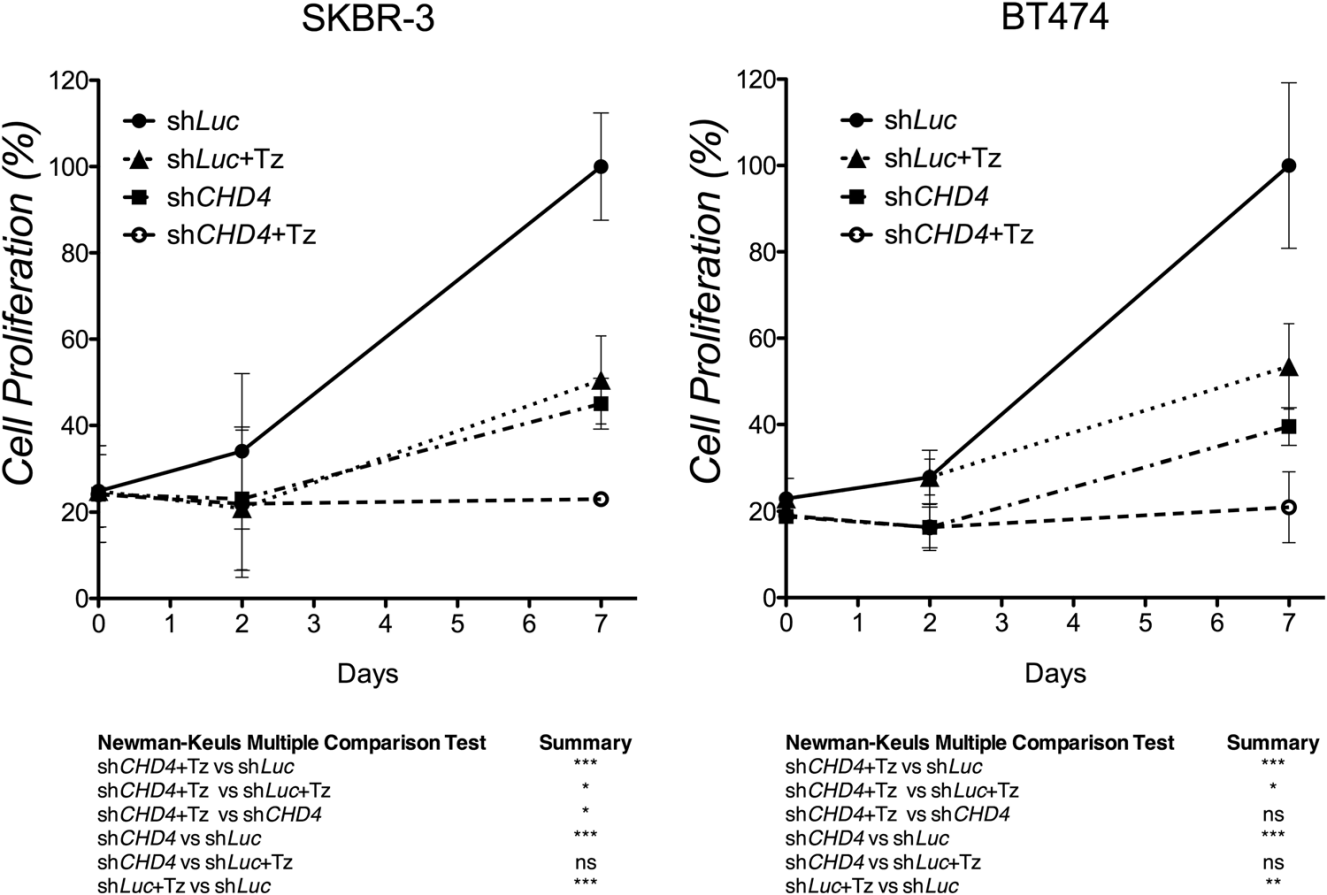
***CHD4* silencing cooperates with Tz in inhibiting survival of ERBB2^+^ breast cancer cells.** SKBR-3 and BT474 cells infected with sh*CHD4* or control sh*Luc* were cultured for 7 days and Tz was administered every 48–72 h. Cell proliferation is expressed as the percentage of the maximum absorbance (570 nm) value obtained after exposure of cultures to MTT for 4 h. Mean values and s.d. (indicated as vertical bars) from three independent replicates are shown. *P<0.05, **P<0.01, ***P<0.001; ns, not significant.

Due to the occurrence of Tz resistance in ERBB2^+^ BC patients, combinatorial anticancer therapies could represent a major advance over single-molecule inhibition. Recently, it has been shown that depletion of *CHD4* sensitizes cancer cells to therapeutic agents (e.g. PARP inhibitors and DNMT inhibitors) in both hematopoietic and solid tumors (Cai et al., 2014; Nio et al., 2015; Sperlazza et al., 2015). Therefore, we hypothesized that the depletion of *CHD4* might cooperate with Tz to reduce proliferation of ERBB2^+^ BC cells. To this end, we transduced SKBR-3 and BT474 cells, both responsive to Tz, with sh*CHD4* or sh*Luc* and then administered Tz every 48 or 72 h for 7 days to evaluate cell number by MTT analysis. As expected, we found that *CHD4* depletion together with Tz significantly inhibited ERBB2^+^ BC cell proliferation compared to control sh*Luc* (alone) in both cell lines (Fig. 6). Interestingly, in SKBR-3 cells the combined treatment is also more effective than the *CHD4* depletion alone (Fig. 6). Collectively, these data suggest that in some ERBB2^+^ expressing cells the depletion of *CHD4* may cooperate with Tz in the inhibition of cell proliferation.

## DISCUSSION

The catalytic core component of the NuRD complex CHD4 has been recently implicated in BC growth and suggested as a novel pharmacological target to block tumor progression (D'Alesio et al., 2016). Moreover, the NuRD complex is implicated in the epigenetic regulation of autophagy (Wang et al., 2013; Wei et al., 2015), which is recognized as a pro-survival process in ERBB2^+^ BC cells resistant to Tz (Vazquez-Martin et al., 2009). In this study, we specifically addressed whether *CHD4* depletion has an impact on ERBB2 signaling pathway and autophagy using two human ERBB2^+^ BC cell lines.

In the present work, as expected on the basis of a previous report (D'Alesio et al., 2016), we found that *CHD4* depletion effectively inhibits cell proliferation of both SKBR-3 and BT474 cells, as evaluated by MTT analysis. Furthermore, we showed by immunoblot analysis that ERBB2 levels are slightly downregulated in *CHD4* silenced cells, likely due to a post-translational mechanism. We also found in these cells an enhancement of ERBB2 Tyr^1248^ phosphorylation. Interestingly, ERBB2 Tyr^1248^ phosphorylation is also induced by Tz and mediates cell growth inhibition (Dokmanovic et al., 2014). Therefore, we suggest that ERBB2 Tyr^1248^ phosphorylation induced by *CHD4* silencing might have an inhibitory effect on the downstream ERBB2 signaling cascade. Consistently, we also found that *CHD4* depletion downregulates PI3K protein levels and the phosphorylation of two key pro-survival and proliferation kinases, AKT and ERK, respectively, by immunoblot analysis. The inhibition of AKT and ERK phosphorylation was further confirmed by a liquid-phase immunoassay-based method. As expected from these results, we found the CDK inhibitor p27^KIP1^, which also mediates cell cycle arrest in Tz-treated cells (Valabrega et al., 2005; Nahta and Esteva, 2006; Le et al., 2005), was dramatically upregulated in *CHD4* silenced cells, as revealed by western blot analysis. Overall, these data point to a growth inhibitory effect of *CHD4* depletion via downregulation of the ERBB2 signaling cascade. However, further studies are needed to reveal how mechanistically loss of *CHD4* affects this cascade.

The role of epigenetic mechanisms in regulating autophagy is an emerging field of study (Baek and Kim, 2017). Interestingly, a body of evidence shows that the NuRD complex plays an important role in the transcriptional regulation of autophagy players (Wang et al., 2013; Wei et al., 2015). Thus, to better understand the relationships between *CHD4* and autophagy in ERBB2^+^ BC cells, we evaluated the expression of two hallmarks of this pathway, LC3 and p62 by immunoblot analysis. We found that *CHD4* silencing caused accumulation of p62, along with a strong increase of the LC3 II/I ratio, suggesting a block of autophagy at late stages. Lysosomes represent the final stage of both the endocytic and autophagic pathways, resulting in the release of breakdown products into the cytosol for subsequent reuse (Pu et al., 2016; Yin et al., 2016). As dysregulated autophagy affects lysosomal functions, we performed immunofluorescence and ultrastructural analysis of *CHD4* depleted and control cells. We demonstrated by immunofluorescence analysis a modest but significant increase of the size of LAMP1 and LAMP2 labeled lysosomes, which suggested an impaired function (Bandyopadhyay et al., 2014). Consistently, transmission electron microscopy analysis showed a striking enlargement of bona fide autolysosomal structures. These findings support our hypothesis of a block in late autophagy resulting from *CHD4* inhibition. We suggest that the repression of autophagy caused by *CHD4* depletion contributes to growth arrest observed in these cells, which is in line with our very recent report though obtained in a different experimental context (D'Alesio et al., 2017).

Combinatorial treatment of ERBB2^+^ BC with Tz and other inhibitors appears as a beneficial approach to improve survival of patients who have failed to previous treatment strategies. Therefore, we wanted to investigate the potential of a combinatorial approach with Tz treatment and *CHD4* depletion in ERBB2^+^ BC cells. When *CHD4* depletion was combined with Tz treatment, we observed a complete block in cell proliferation, while the single inhibition/treatment only achieved a decrease of cell proliferation, evaluated by MTT analysis. In particular, the combined treatment resulted in a statistically significant reduction of cell proliferation versus the Tz treatment alone in both cell types, whereas significance was reached by the combined treatment versus CHD4 depletion only in SKBR-3 cells.

In conclusion, these results warrant further studies in animal models with the aim to evaluate the effectiveness of the combinatorial treatment of Tz with putative pharmacological inhibitors of *CHD4* in the inhibition of ERBB2^+^ BC development and/or progression.

## MATERIALS AND METHODS

### Cell culture and *CHD4* silencing

BC cell lines SKBR-3 and BT474 were obtained from Banca Biologica and Cell Factory in IRCCS Ospedale Policlinico San Martino belonging to the European Culture Collection's Organization. Cells were cultured in complete medium [DMEM high glucose supplemented with 10% heat inactivated fetal bovine serum, 1% glutamine and penicillin and streptomycin (Euroclone s.p.a., Milan, Italy)], at 37°C in a humidified atmosphere containing 5% CO_2_.

*CHD4* silencing was performed as recently described (D'Alesio et al., 2016). The shRNAs targeting *CHD4* were used as pools of two distinct shRNAs. An shRNA targeting the firefly *Luciferase* (*Luc*) mRNA was used as negative control. Complete sequences of *CHD4* and *Luc* shRNAs are provided in Table S2. Silencing efficacy was measured using RTqPCR. This analysis demonstrated that ERBB2^+^ BC cells transduced with sh*CHD4* expressed significantly less than 45% and 30% *CHD4* mRNA compared to sh*Luc* controls in both SKBR-3 and BT474 cell lines up to 7 days after transduction, respectively (Fig. S1).

### Cell proliferation assay

SKBR-3 and BT474 cells were plated in 24-well plates in complete medium (triplicate of SKBR-3 35,000 cells/well and BT474 55,000 cells/well). Cell proliferation was measured at different time points using the 3-(4,5-dimethylthiazol-2yl)-2,5-diphenyltetrazolium bromide (MTT) colorimetric assay.

Tz (Genentech-Roche, South San Francisco, CA, USA) was dissolved with saline solution with 0.9% NaCl in a stock concentration of 21 mg/ml, donated by the pharmacy (UFA-Unità Farmaci Antiblastici) of the IRCCS Ospedale Policlinico San Martino. Tz was used at a concentration of 5 µg/ml for SKBR-3 and 0.21 µg/ml for BT474. Control cells were cultured with human IgGs at the same concentrations used for Tz. Both Tz and IgGs were administered every 48 or 72 h for 7 days.

### Antibodies

All primary antibodies used in this study are listed in Table S3.

### Western blot analysis

Transduced SKBR-3 and BT474 cells were cultured for 48 h and lysed using lysis buffer (Hepes pH 7.4 20 mM, NaCl 150 mM, 10% Glycerol, 1% Triton X-100) with protease inhibitors cocktail Complete (Roche Applied Science, Penzberg, Germany) and sodium orthovanadate or Phostop (Roche) used both as phosphatase inhibitors. Protein quantification was performed using Bradford protein assay (BioRad) and protein extracts were resolved on SDS-polyacrylamide gel electrophoresis (Invitrogen). Gels were then blotted onto nitrocellulose (GE Healthcare, Little Chalfont, UK) membranes and probed with appropriate primary antibodies (Table S3). Secondary antibodies were horseradish peroxidase-conjugated: anti-mouse or rabbit (Thermo Fisher Scientific) and anti-goat (Santa Cruz Biotechnology Inc.) and proteins detection was performed with ECL Detection Reagent (GE Healthcare) according to manufacturer's protocol. ECL signals were detected and measured by the Uvitec Chemiluminescence Imaging System and NineAlliance software (Uvitec Ltd., Cambridge, UK). ECL signals were detected and measured by the Uvitec Chemiluminescence Imaging System and ImageJ software (Bandyopadhyay et al., 2014).

### Alphaplex assay

Transduced SKBR-3 and BT474 cells were cultured for 48 h and lysed with Lysis Buffer (PerkinElmer, Waltham, MA, USA). Samples were then processed using the Alpha SureFIre Ultra Multiplex kits (PerkinElmer) for phospho AKT and ERK1/2. Protein levels were measured with EnVision 2105 Multimode Plate Reader (PerkinElmer) and analyzed according to manufacturer's protocol (https://www.perkinelmer.com/lab-solutions/resources/docs/MAN_Alpha_SureFire_Multiplex_HV_pAKT_SingleKit.pdf).

### Immunofluorescence analysis

Transduced SKBR-3 and BT474 cells were cultured for 48 h, fixed in 3% paraformaldheyde (PFA) in phosphate-buffered saline (PBS) pH 7.4 and then quenced with 30 mM NH4Cl. Subsequently, cells were permeabilized with 0.2% saponin and incubated for 1 h at room temperature with anti-LAMP1 and anti-LAMP2 antibodies to reveal lysosomes. The secondary antibodies were incubated for 30 min in 0.2% saponin/PBS: Alexa488-conjugated donkey anti-mouse or Alexa456-conjugated donkey anti-mouse (Thermo Fisher Scientific). The coverslips were mounted using Prolong Gold with DAPI and anti-fading reagent (Thermo Fisher Scientific). Image acquisition and real time deconvolution was performed with an Axio Imager A2M microscope equipped with an Apotome module for structured illumination epifluorescence (Carl Zeiss, Jena, Germany). Quantification of LAMP1 and LAMP2 lysosome size was performed by using the object analyzer advanced tool of Huygens Professional version X11 (http://svi.nl) (Scientific Volume Imaging, The Netherlands).

### Transmission electron microscopy

Transduced SKBR-3 and BT474 cells were seeded and cultured on glass chamber slides (Lab-Tek 177380, Nalge Nunc int., Rochester, NY, USA). Cells were washed out in 0.1M cacodylate buffer and fixed in 0.1M cacodylate buffer containing 2.5% glutaraldehyde (Electron Microscopy Science, Hatfield, PA, USA), for 1 h at room temperature. Samples were postfixed in osmium tetroxide for 2 h and 1% uranyl acetate for 1 h. Cells were next dehydrated through a graded ethanol series and flat embedded in resin (Poly-Bed; Polysciences, Inc., Warrington, PA, USA) for 24 h at 60°C. Ultrathin sections (50 nm) were cut parallel to the substrate, stained with 5% uranyl acetate in 50% ethanol and observed with a CM10 electron microscope (Philips, Eindhoven, The Netherlands). Digital images were captured with a Megaview II camera. Analysis of the size of morphologically AL was assessed in 10 cells for each treatment, as recently reported (D'Alesio et al., 2017; Thellung et al., 2018). The diameter of each organelle was measured with the iTEM software package (Olympus-SYS; Olympus Corporation, Shinjuku, Tokyo, Japan) and plotted as box plot.

### RNA extraction and real-time qPCR

RNA was extracted using Trizol reagent (Thermo Fisher Scientific), cDNA was synthesized and RT qPCR was performed in quadruplicate using 1×IQTM SybrGreen SuperMix and CFX apparatus (Biorad). The relative quantity of target mRNA was calculated by the comparative Cq method using glyceraldehyde 3-phosphate dehydrogenase (*GAPDH*) as housekeeping gene (Fwd: 5′-ACCCACTCCTCCACCTTTGACG-3′; Rev 5′- CTCTTGTGCTCTTGCTGGGGCTG-3′), and expressed as fold induction with respect to controls (Pfaffl, 2001). *CHD4* primer pairs (Fwd 5′- TGGCCCAGTATGTGGTACG −3′; Rev 5′- CCTGTTTAATGATTTCCCGTTC −3′) were purchased from Sigma-Aldrich. *ERBB2* primer pairs (Fwd 5′- CAACTGCACCCACTCCTGT −3′; Rev 5′- GCAGAGATGATGGACGTCAG −3′) were synthesized by Tib MolBiol s.r.l. custom oligonucleotides synthesis service (Genova, Italy). Amplification conditions were 3 min at 95°C followed by 5 s at 95°C and 30 s at 60°C for 40 cycles.

### Statistical analyses

Statistical analyses were performed using Prism (GraphPad Software, La Jolla, CA, USA).

All measurements here reported are presented as mean±standard deviations (s.d.). For cell survival assay (sh*Luc* versus sh*CHD4*), for Alphaplex analysis, and qPCR analysis we used a two-tailed distribution Student's *t*-test. For ultrastructural studies, we used *t* Student test plus post-hoc Mann–Whitney comparison test. For cell survival assay (sh*Luc* versus sh*CHD4* ^+^/- Tz administration), we used one-way ANOVA plus post-hoc Newman-Keuls multiple comparison test. Mean differences were considered statistically significant (*P-*value) at *P*<0.05.

## Supplementary Material

Supplementary information
