# Supplementary material for: The chromodomain helicase CHD4 regulates ERBB2 signaling pathway and autophagy in ERBB2+ breast cancer cells
Source: Biol Open. 2019 Apr 9;8(4):bio038323. doi: 10.1242/bio.038323 (PMC6504000; doi:10.1242/bio.038323)
Supplement: Supplementary information [file biolopen-8-038323-s1.pdf]

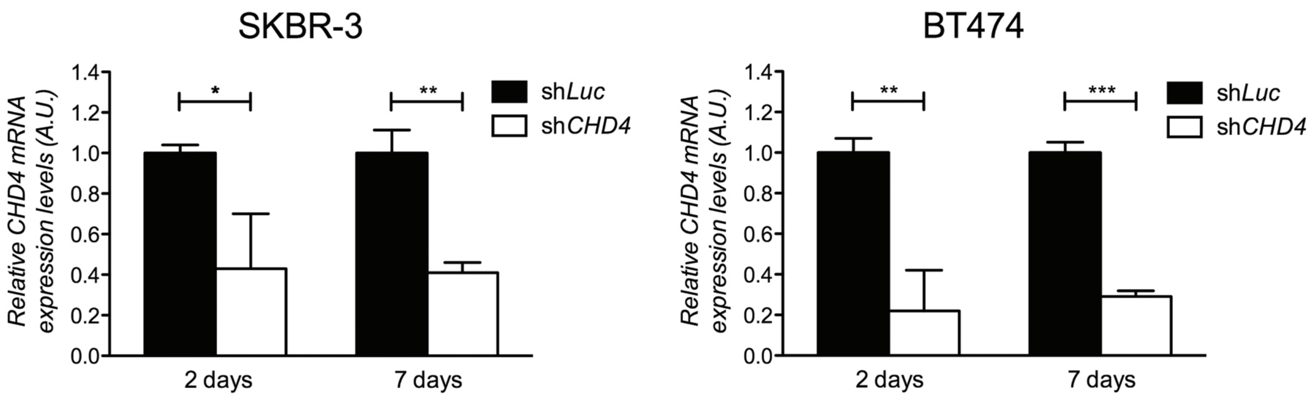

Figure S1. CHD4 silencing efficacy in ERBB2+ BC cells. SKBR-3 and BT474 cells transduced with sh*CHD4* or control sh*Luc* were cultured for 48 hours (2 days) or 7 days and subsequently RNA was extracted. *CHD4* mRNA expression levels were detected by qPCR analysis and are expressed as arbitrary units (A.U.). Mean values and s.d. (indicated as vertical bars) from three independent replicates are shown. *P*<0.05 (*), *P*<0.01 (**), *P*<0.001 (***).

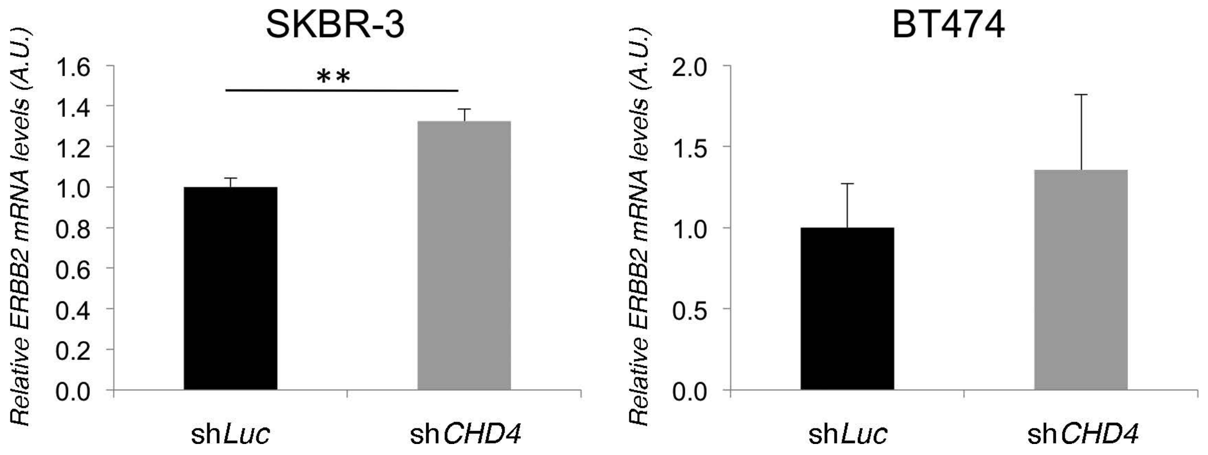

Figure S2. *ERBB2* mRNA expression levels in SKBR-3 and BT474 cells. ERBB2+ BC cells infected with sh*CHD4* or control sh*Luc* were cultured for 48 hours and subsequently RNA was extracted. *ERBB2* mRNA expression levels were detected by qPCR analysis and are expressed as arbitrary units (A.U.). Mean values and s.d. (indicated as vertical bars) from three independent replicates are shown. *P*<0.01 (**).

Biology Open • Supplementary information

Table S1. Relative expression levels of the indicated proteins, or ratio between the indicated phosphorylated/total protein isoforms, expressed in arbitrary units after immunoblot analysis as indicated in materials and methods.

Protein or ratio of protein isoforms	Average shCHD4 vs shLuc		Experiment #1 shCHD4 vs shLuc		Experiment #2 shCHD4 vs shLuc		Experiment #3 shCHD4 vs shLuc	
	SKBR-3	BT474	SKBR-3	BT474	SKBR-3	BT474	SKBR-3	BT474
CHD4	0.4	0.4	0.3	0.1	0.2	0.5	0.8	0.6
pERBB2	7.9	2.8	1.8	1.6	17.6	5.3	4.4	1.5
ERBB2	0.8	0.6	0.6	0.7	0.9	0.2	0.9	0.8
PI3K	0.6	0.5	0.7	0.7	0.5	0.1	0.6	0.7
pAKT/AKT	0.3	0.7	0.1	0.6	0.7	0.7	0.1	0.9
pERK/ERK	0.7	0.8	0.8	0.8	0.9	0.9	0.5	0.8
P27	9.1	6	13	12	3.3	3.1	11	2.8
P62	3.6	3.1	3	5.5	6.2	2	1.5	1.7
LC3II/LC3I	6.6	4.1	2.4	6	15.7	4.7	1.6	1.7

Table S2. shRNAs sequences used to transduce ERBB2+ breast cancer cells

	Sequences 5' -> 3'
shLUC	CAAATCACAGAATCGTTGTAT
shCHD4 1	GCGGGAGTTTAGTACTAATAA
shCHD4 2	CCTCGAGTGAGGGTGATGATT

Biology Open • Supplementary information

Table S3. Antibodies used in the study*.

Target protein	Source	Catalog number
AKT	Cell Signaling	9272
CHD4	Abcam	ab70469
ERK 1/2	Santa Cruz Biotechnology	sc-135900
ERBB2	Santa Cruz Biotechnology	sc-284
LAMP1	Developmental Studies Hybridoma Bank	H4A3
LAMP2	Developmental Studies Hybridoma Bank	H5C6
LC3	Novus Biologicals	NB100-2220
p27kip1	Cell Signaling	3686
p62	Novus Biologicals	H00008878-M01
PI3K	Cell Signaling	C73F8
phospho-AKT (Ser473)	Cell Signaling	9271
phospho-ERK1/2 (Thr 202/Tyr 204)	Santa Cruz Biotechnology	sc-16982
phospho-ERBB2 (Tyr 1248)	Cell Signaling	2247
Tubulin	Sigma	T5168
Vinculin	Sigma	V9131

* All antibodies were used at the final concentration recommended by the supplier.
